# Sight and sound out of synch: Fragmentation and renormalisation of audiovisual integration and subjective timing

**DOI:** 10.1016/j.cortex.2013.03.006

**Published:** 2013-11

**Authors:** Elliot D. Freeman, Alberta Ipser, Austra Palmbaha, Diana Paunoiu, Peter Brown, Christian Lambert, Alex Leff, Jon Driver

**Affiliations:** a City University London, United Kingdom; b John Radcliffe Hospital, Oxford University, United Kingdom; c St George's, University of London, United Kingdom; d Institute of Cognitive Neuroscience, University College London, United Kingdom

**Keywords:** Audiovisual integration, Psychophysics, Individual differences, Illusions, Sensory timing

## Abstract

The sight and sound of a person speaking or a ball bouncing may seem simultaneous, but their corresponding neural signals are spread out over time as they arrive at different multisensory brain sites. How subjective timing relates to such neural timing remains a fundamental neuroscientific and philosophical puzzle. A dominant assumption is that temporal coherence is achieved by sensory resynchronisation or recalibration across asynchronous brain events. This assumption is easily confirmed by estimating subjective audiovisual timing for groups of subjects, which is on average similar across different measures and stimuli, and approximately veridical. But few studies have examined normal and pathological individual differences in such measures.

Case PH, with lesions in pons and basal ganglia, hears people speak before seeing their lips move. Temporal order judgements (TOJs) confirmed this: voices had to *lag* lip-movements (by ∼200 msec) to seem synchronous to PH. Curiously, voices had to *lead* lips (also by ∼200 msec) to maximise the McGurk illusion (a measure of audiovisual speech integration). On average across these measures, PH's timing was therefore still veridical. Age-matched control participants showed similar discrepancies. Indeed, normal individual differences in TOJ and McGurk timing correlated *negatively*: subjects needing an auditory lag for subjective simultaneity needed an auditory lead for maximal McGurk, and vice versa. This generalised to the Stream–Bounce illusion. Such surprising antagonism seems opposed to good sensory resynchronisation, yet average timing across tasks was still near-veridical.

Our findings reveal remarkable disunity of audiovisual timing within and between subjects. To explain this we propose that the timing of audiovisual signals within different brain mechanisms is perceived relative to the average timing across mechanisms. Such renormalisation fully explains the curious antagonistic relationship between disparate timing estimates in PH and healthy participants, and how they can still perceive the timing of external events correctly, on average.

## 1 Introduction

When a person speaks, we usually expect to hear their voice at the same time as seeing their lips move. Furthermore, if we watch their lips, it often helps us to hear their voice better, via ‘speechreading’ (Sumby and Pollack, 1954). Two distinct kinds of processes are implied by such observations: synchronisation and integration. Firstly, we are sensitive to when auditory and visual events are occurring at the same time (Alais and Carlile, 2005; King, 2005; Kopinska and Harris, 2004; Sugita and Suzuki, 2003). Secondly, the ability to benefit from the combination of modalities, as in speechreading, requires that auditory and visual information be brought together in the brain and integrated. So automatic and compelling is such integration that artificial mismatches between sound and vision can easily induce illusory changes in the perceived location, timing or actual interpretation of the stimuli (Bertelson and Radeau, 1981; Howard and Templeton, 1966; McGurk and MacDonald, 1976; Witkin et al., 1952).

It is easy to take for granted that audiovisual events are always synchronised and integrated correctly. But here, we present the first ever confirmed case of a patient (PH) who hears peoples' voices *before* he sees their lips move. Testing this individual in comparison with neurologically healthy participants gave us the unique opportunity to address two issues: Firstly, we ask whether PH's auditory leading phenomenon is selective for subjective synchrony or whether his audiovisual integration is also affected. This addresses a current debate over whether optimal integration depends on achieving *subjective* synchrony, or whether integration obeys independent temporal constraints (Arnold et al., 2005; Martin et al., 2013; Munhall et al., 1996; Soto-Faraco and Alsius, 2007, 2009; van Wassenhove et al., 2007). Secondly, PH's pathological desynchronisation might provide insight into the deeper question of how (or indeed whether) sensory synchronisation is normally achieved, which has long perplexed neuroscientists and philosophers (Dennett and Kinsbourne, 1995; Harris et al., 2008; Keetels and Vroomen, 2012; Spence and Squire, 2003; Vroomen and Keetels, 2010; Zeki and Bartels, 1998). We consider this issue first.

### 1.1 Multisensory synchronisation

The problem of synchronisation is exemplified by the maxim known as Segal's law: ‘*With one clock you always know the time; with two you are never sure*’. Does the brain also have multiple clocks, and if so, does this create a similar uncertainty? There are many multimodal convergence zones in the brain (Bushara et al., 2001; Cappe et al., 2009; Driver and Noesselt, 2008; Ghazanfar and Schroeder, 2006; Macaluso and Driver, 2005; Meredith et al., 1987; Stevenson et al., 2010), and to get there, auditory and visual signals must traverse different routes and distances, thus most likely arriving at different times (Arnold et al., 2001; Aschersleben and Prinz, 1995; Halliday and Mingay, 1964; Moutoussis and Zeki, 1997; Stone et al., 2001). Consequently each area will have different information about when visual and auditory events occurred (Scharnowski et al., 2013). This entails a ‘multiple-clocks’ uncertainty for knowing the absolute and relative timing of external events.

Despite such systemic and intrinsic asynchrony, subjects still often recognise when auditory and visual sources are approximately synchronous (Harris et al., 2008), at least for proximal if not always for distal stimuli (Alais and Carlile, 2005; Arnold et al., 2005; Heron et al., 2007; King, 2005; Kopinska and Harris, 2004; Stone et al., 2001; Sugita and Suzuki, 2003; Vroomen and Keetels, 2010). Shifts in subjective simultaneity following adaptation to asynchrony are consistent with the existence of mechanisms functioning at least locally to resynchronise temporal discrepancies between modalities (Fujisaki et al., 2004; Hanson et al., 2008; Miyazaki et al., 2006; Yamamoto et al., 2012). However, individuals differ widely with respect to the objective audiovisual asynchrony which they perceive as subjectively synchronous (the Point of Subjective Simultaneity – PSS; Stone et al., 2001). This may depend intrinsically on the time for neural conduction and processing of signals, which may differ between stimuli and individuals (Arnold et al., 2001; Aschersleben and Prinz, 1995; Halliday and Mingay, 1964; Moutoussis and Zeki, 1997; Stone et al., 2001), though attentional biases may also account for some apparent individual differences in multisensory timing (Spence and Parise, 2010; Spence et al., 2001). Furthermore, even within the same subjects given the same stimuli, different tasks produce uncorrelated estimates of PSS (van Eijk et al., 2008) though such variations may depend on strategic variables (García-Pérez and Alcalá-Quintana, 2012; Schneider and Bavelier, 2003; van Eijk et al., 2008). Thus synchronising mechanisms, if they exist (Zeki and Bartels, 1998), may not function perfectly.

If there were a single specialised mechanism for multisensory synchronisation, one might expect to find individuals for whom different modalities have been chronically desynchronised following a brain trauma. Loss of acuity for temporal order has been observed following temporal lobectomy (Sherwin and Efron, 1980), but the lack of selective impairments in temporal processing is inconsistent with the notion of a unitary specialised mechanism underlying timing perception (Wiener et al., 2011). Indeed, there is only one previously reported case of apparently acquired sensory desynchronisation (Hamilton et al., 2006). Hamilton et al. (2006) described patient AWF who claimed to experience ‘*a perceived temporal mismatch*’ (Abstract). However they did not specify whether vision actually preceded or lagged audition, and did not formally quantify the temporal mismatch using objective measures, for example by measuring performance across a range of audiovisual asynchronies. Thus to date, evidence that sensory synchronisation can be pathologically impaired rests largely on AWF's subjective report, which is not very specific.

### 1.2 Dependence of integration on synchronisation

While investigations of synchronisation have typically focused on temporal relationships between modalities (e.g., Harris et al., 2008), the multiple-clocks problem also logically applies more generally between different processes. Here we consider two such notional processes, supporting subjective temporal judgements versus those that serve to integrate inputs from different modalities. We ask whether sound and vision are optimally integrated when they are subjectively synchronous. These processes are not logically the same, and evidence from functional brain imaging suggests they are supported by distinct brain mechanisms (Bertini et al., 2010; Miller and D'Esposito, 2005; Stevenson et al., 2010). Given such separation, and the ‘multiple-clocks’ problem which that entails, any evidence of dependence of integration on synchronisation could be indicative of synchronising mechanisms operating between distinct cognitive processes, not just between modalities as a whole.

It seems intuitive that such *unity* of timing across processes should be achieved. Such an intuition might be based on the assumption that single physical events should be associated with a unitary percept (Welch and Warren, 1980). It might indeed be surprising if we consciously perceived different aspects of the same event as occurring at different times (though in some cases it seems we do; Arnold et al., 2001; Moutoussis and Zeki, 1997). Evidence suggests that the brain does actively strive to maintain synchrony across processes. For example in the ‘unity effect’, stimuli which are readily integrated (such as meaningful speech sounds and lip-movements) tend to be judged as synchronous even if they are actually not (Vatakis and Spence, 2007). Conversely, integration may depend on a prior decision about the temporal correspondence of auditory and visual streams. For example, in the classic McGurk illusion (McGurk and MacDonald, 1976), the combination of a voice saying /ba/ and a face mouthing [ga] often results in hearing the syllable /da/, while auditory /da/ with visual [ba] can sound like /ba/, but such visual interference declines (on average) with increasing asynchrony between voice and lips (Munhall et al., 1996; Soto-Faraco and Alsius, 2007, 2009; van Wassenhove et al., 2007). Similarly for non-speech stimuli, we are more likely (on average) to perceive two balls as bouncing off each other when their collision is accompanied simultaneously by a sound, compared to when these auditory and visual events are asynchronous (Sekuler et al., 1997). Though such findings demonstrate dependence of integration on synchrony, on average across participants, its critical dependence on individuals' own subjective synchrony has not been examined to date.

The above positive evidence suggests that the brain actively benefits from, and actively strives for subjective unity across its different process. But however desirable, a unitary percept may not always be achieved. Some observations appear to challenge the intuitive dependence of multisensory integration on audiovisual synchronisation (Spence and Ngo, 2012). For example in the McGurk effect, Soto-Faraco and Alsius (2007, 2009) used a dual-task paradigm to measure McGurk interference and subjective synchrony as a function of audiovisual asynchrony. They found that illusory McGurk percepts were often reported even for audiovisual stimuli that could be reliably identified as asynchronous (on average across participants). Such ‘*dual perception*’ of good lip-voice integration despite a detectable audiovisual asynchrony hints that unity can be violated, and thus in principle measures of subjective integration and synchronisation might be based on their own stimulus inputs and correspondence mechanisms rather than shared or synchronised mechanisms. Some doubt remains about this however, because integration and synchronisation judgements still centred on similar near-veridical asynchronies (on average), and thus could still be subject to common synchronising mechanisms. Furthermore, any apparent differences between the measures might just reflect different criteria for deciding whether two asynchronous events from different modalities should be integrated or segmented, compared to when deciding whether the two events are synchronous or asynchronous. The mismatch between measures was also small, though note that these measures were averaged across observers, which might conceal the true extent to which optimal timing may differ between mechanisms within individuals.

Neuropsychological studies might contribute to this debate if cases could be found where brain lesions result in selective impairment of either synchronisation or integration, or joint impairment of both together. A case of the latter kind seems to be reported by Hamilton et al. (2006), where the ‘temporal mismatch’ experienced by patient AWF coincided with an eliminated McGurk effect for veridically synchronous stimuli. However Hamilton et al. did not test McGurk under different conditions of audiovisual asynchrony. Thus the critical evidence for true interdependence of synchronisation and integration functions was lacking, which would have been provided if the McGurk effect had been reinstated in AWF, for subjectively simultaneous stimuli.

### 1.3 The present study

From the above review it may be concluded that the question of how, or indeed whether, the brain can minimise discrepancies in timing between modalities and between cognitive processes, has not yet been satisfactorily resolved. Critical insights may be gained by studying individual differences between measures probing synchronisation and integration, and comparing natural variations in these measures with those acquired following brain injury.

In particular, we can examine (1) whether PH is an example of a categorical breakdown of putative unifying mechanisms, or whether his lesions have merely shifted him along a continuum of disunity, where we may also find ourselves. We therefore ask, how unusual is PH (Experiment 1)? If highly abnormal, he could be ‘the exception that proves the rule’, that unity and synchrony are normally achieved in individuals (albeit with inaccuracies). But exceptions can also ‘prove’ rules wrong. Our evidence, of large discrepancies between our two measures in PH and surprisingly also in normal subjects, suggests that asynchrony and disunity may rule instead.

We can also ask (2) whether PH's acquired subjective asynchrony is specific to perception of audiovisual temporal order or whether this affects the temporal tuning of audiovisual integration, and also how closely measures of integration correspond with measures of synchrony, within normal individuals. We assess integration in the McGurk effect (Experiment 2) and the Stream–Bounce illusion (Experiment 3). If multimodal integration and synchronisation of speech stimuli are based on dependent mechanisms, it seems straightforward to predict that individual differences for our two measures will correlate positively. Alternatively, a null correlation seems intuitively likely if these mechanisms were fully independent. We find neither. Our counterintuitive results call for a revised understanding of how the brain solves the multiple-clocks problem, which we propose.

Our study simply replicated the dual-task paradigm of Soto-Faraco and Alsius (2007) with PH and normal controls. On each trial, we presented brief movies with a range of audiovisual asynchronies. There then followed two tasks, temporal order judgement (TOJ) and phoneme discrimination, to obtain two concurrent measures of the audiovisual asynchrony that (1) is perceived as synchronous, and (2) induces maximal integration, as measured by the strength of the McGurk illusion. We then analysed individual differences on these measures rather than just average performance.

As PH's phenomenology is of a distinct temporal order, of lips lagging voices, TOJ was chosen to probe his subjective report as directly as possible. We were also concerned that the alternative paradigm, Simultaneity Judgement, might be performed heuristically on the basis of the quality of speech integration, and thus our measure of subjective timing in PH and control subjects might have been confounded by changes in integration as a function of asynchrony.

Before reporting the methods and results of our experiments we first provide detailed documentation of case PH.

## 2 PH case study

PH, a retired pilot aged 67, first experienced auditory leading while watching television. He initially suspected poor dubbing, but then later noticed the same phenomenon in conversations with people. After seeking medical advice at his workplace, he was referred to Professor Peter Brown at his Queen Square neurology clinic, where we recruited him for this research. He also reports perceiving the sound of his own voice before the proprioception of his corresponding mouth and jaw movements. The onset seems to have been abrupt, not accompanied by any other symptoms, and initially progressing slowly but now stable according to his subjective reports, though becoming temporarily more intense when fatigued. He also reported experiencing difficulty in speech comprehension in noisy environments, though attributes this to tinnitus. In November 2007 he had surgery to treat pericarditis, and in 2008 he had developed generalised myasthenia gravis [anti-acetylcholine (ACh) receptor antibody and electromyography (EMG) positive]. His current complaint came on 2–3 months after the onset of the myasthenia, however it is unknown to what extent these phenomena are related (Keesey, 1999).

A routine neurological examination revealed no abnormalities. There was no evidence of fatiguability. Mild hearing loss for high frequencies was observed using audiometry. Performance in a standard battery of neuropsychological tests (Table 1) revealed generally high functioning with no specific functional impairments. He showed above average Wechsler intelligence quotient (IQ) (The Psychological Corporation, 1999) and near-perfect performance on tests of everyday attention (Robertson, 2006), and the Visual Object and Space Perception Battery (Warrington and James, 1991), with the sole exception of silhouette identification (19/30). Sentence repetition (Spreen and Benton, 1969), performed while the speaker's face was hidden from view, was perfect and immediate.

### 2.1 Imaging

**Fig. 1:**
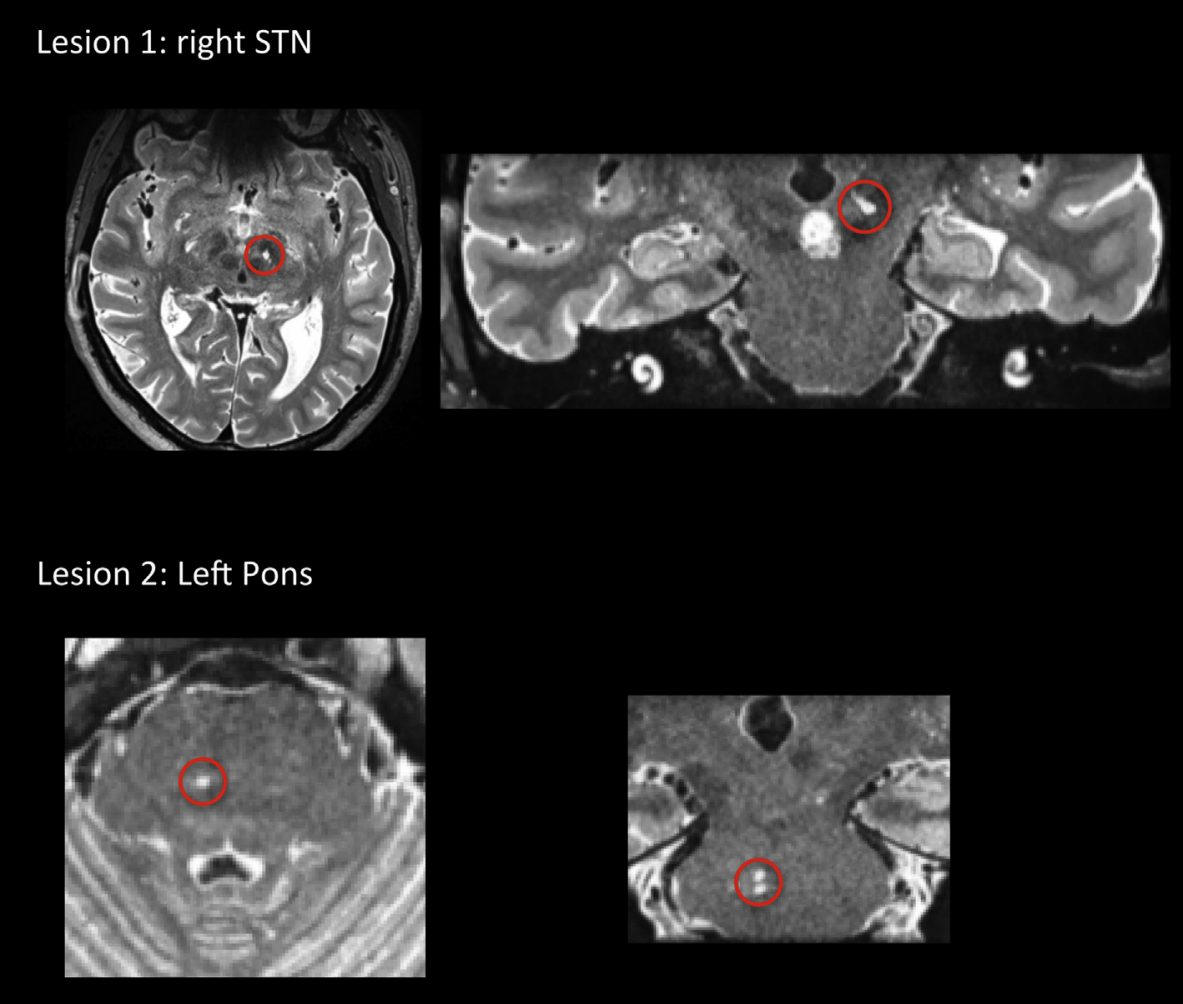
T2 weighted images of both lesion sites, outlined in red.

High resolution magnetic resonance imaging (MRI) (500 μm^3^) revealed two lesions. Lesion 1 was located in superior mesencephalon, at the left anterio-medial tip of the subthalamic nucleus (11.5 mm left and 16.8 mm posterior to the anterior commisure). Total lesion volume was 42 mm^3^. Lesion 2 was located in mid-brainstem within the right dorso-medial pontine nucleus at the level of middle cerebellar peduncle around the exit of the trigeminal nerve (see Fig. 1). These were considered likely to represent small established lacunar infarcts. There was no evidence of an acute ischaemic lesion or microhaemorrhages.

Diffusion tensor imaging (DTI) was undertaken using images from healthy subjects, to identify brain regions which are connected to the lesion sites (see Supplementary Methods S1 and Supplementary Figure 1). Results indicated that lesion 1 had ipsilateral projections predominately into the motor cortico-striato-pallido-thalamic-cortical relay loop, and a small projection with the Orbito-Frontal relay loop. Cortical projections were consistent with Limbic subthalamic nucleus (STN) (Lambert et al., 2012). Lesion 2 lay along the olivo-collicular pathway (Supplementary Figure 1), with largely ipsilateral projections to inferior colliculus and extending down to the medial territory of the peri-olivary nucleus. There was also a possible involvement of the tectopontine pathway. This second lesion may be associated with the early auditory system. Both regions have been implicated in crossmodal interactions (Halverson and Freeman, 2010; Kolomiets et al., 2001), and in event timing (Teki et al., 2011).

## 3 Methods

### 3.1 Healthy participants

Experiment 1 had 10 participants similar in age to PH (59–74 years, mean 65, standard deviation – SD 5). Experiment 2 had 27 neurologically healthy young subjects (18–28 years, mean 22), and included the results from the older age-matched controls. Data from four further participants were excluded, due to poor performance, resulting in implausible estimates of subjective timing >300 msec asynchrony, outside the typical range for multisensory integration (Vatakis et al., 2008; Vatakis and Spence, 2007) and indicative of poor quality data and unreliable function fits. Experiment 3 (testing the Stream–Bounce illusion) had 24 participants aged 18–24, excluding two others who reported no ‘bounce’ illusion. All participants were naïve to the specific aims of this study. Participants received a monetary reward. Procedures were approved by the local Psychology ethics committee.

### 3.2 Apparatus and stimuli

Laboratory apparatus comprised an Apple Mac Mini, with Labtec speakers positioned either side of a 17" Sony HMD-A420 cathode ray tube (CRT) display, viewed in darkness from 70 cm. Mobile apparatus for older participants and PH comprised a Sony Vaio SZ1XP PC with built-in speakers and 13.3" liquid crystal display (LCD) display, viewed from approximately 57 cm. In both cases video mode was 1200 × 800 with a 60 Hz refresh rate. Subjects responded using the cursor keys on the standard keyboard.

**Fig. 2:**
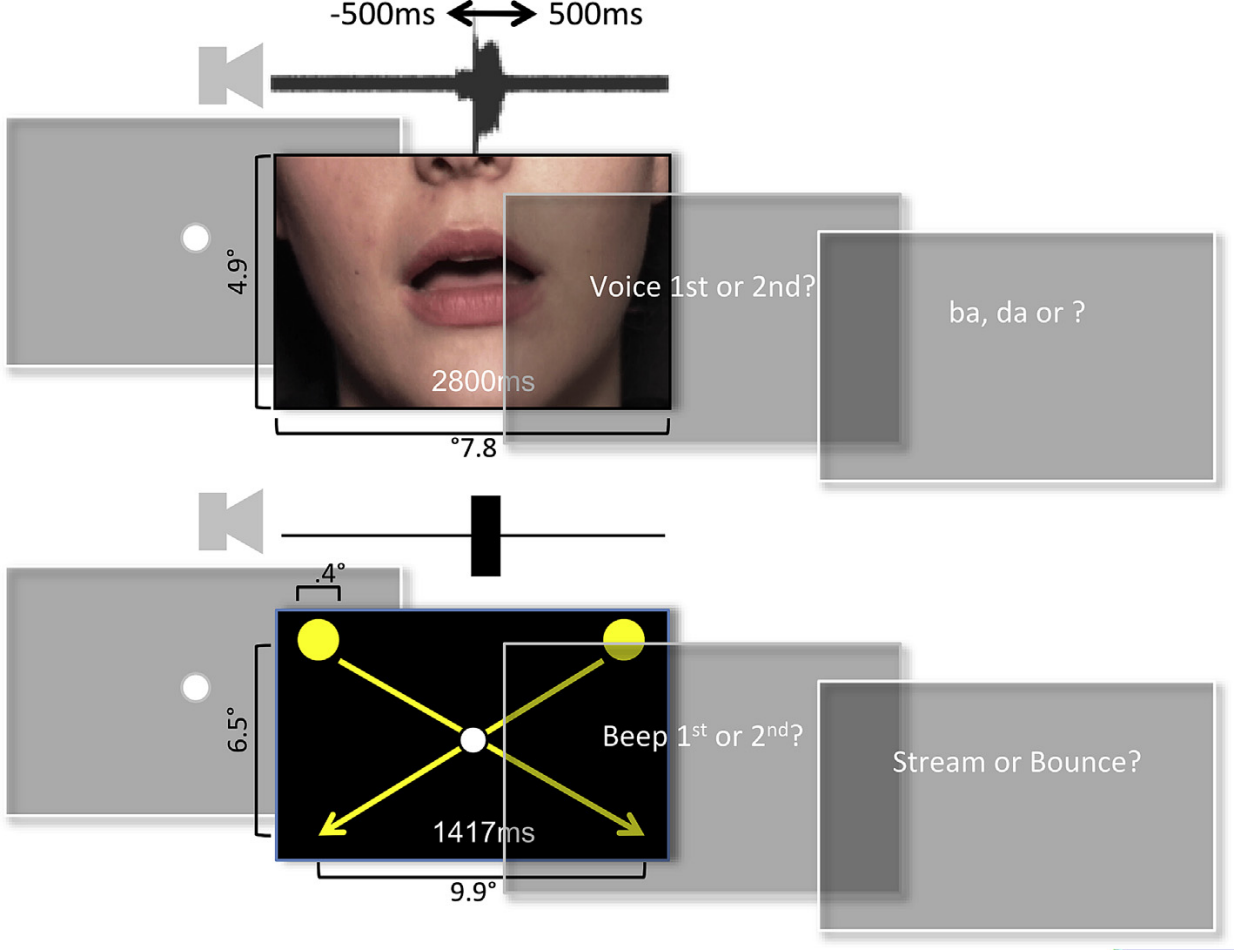
Trial sequence and stimuli for McGurk (top row) and Stream–Bounce illusions (bottom).

McGurk stimuli were based on Soto-Faraco and Alsius (2007), which were kindly provided by the authors (see Fig. 2 for dimensions, and Supplementary Video). Auditory /ba/ and /da/ phonemes (with white noise at 15% of maximum amplitude) were combined with visual lip-movements for [ba], [da] and [ga]. The two incongruent pairings for eliciting the McGurk effect were /ba/ + [ga] = ‘da’ and /da/ + [ba] = ‘ba’ or ‘bda’. The other two ‘congruent’ pairings /ba/ + [ba] and /da/ + [da] tend to be heard correctly. Background was set to the average red green blue (RGB) value across all pixels and frames. For the Stream–Bounce experiment, visual stimuli were two yellow circular at maximum contrast on a black background. Each moved from positions left and right above fixation, via the central fixation point, to opposite positions below fixation (see Fig. 2 for dimensions, and Supplementary Video). Animations were accompanied by a 400 Hz tone of 100 msec duration, with the same manipulation of asynchrony as for the McGurk stimuli. Movies were followed by 9 pt white text prompting responses, displayed centrally.

The following are the supplementary videos related to this article:

Video 1 McGurk stimulus demo: Four combinations, played consecutively: 1. Auditory /ba/ with visual [ba]: congruent. 2. Auditory /ba/ with visual [ga] (incongruent: McGurk effect sounds like “da”). 3. Auditory /da/ with visual [ba] (incongruent: McGurk effect sounds like “ba”). 4. Auditory /da/ with visual [da]: congruent.

Video 2 Stream–bounce stimulus demo. Two examples, played consecutively: 1. Beep simultaneous with visual collision. 2. Beep lags visual collision by 150 msec.

We also tested PH with various biological and/or non-speech stimuli. Finger-click movies, of 3000 msec duration, showed a hand with the middle finger clicking against the thumb. Sequences began with either the hand open (to provide predictive information) or closed. For scrambled-speech stimuli, the soundtrack from the original McGurk stimuli was passed through a three-channel noise vocoder using Praat software (version 5.1.21, http://www.praat.org), rendering the speech unintelligible but without affecting the spectral composition of the sound or the temporal sequence of amplitude modulations. The video sequence remained the same. Non-biological stimuli comprised a white square (1.67 ° on each side) on a black background, presented for 200 msec and paired with a white-noise burst of 200 msec duration.

### 3.3 Design

Except where specified we used a dual-task paradigm (Soto-Faraco and Alsius, 2007) (Fig. 2) to obtain two concurrent measures of the audiovisual asynchrony that is (1) perceived as synchronous, and (2) optimal for maximum audiovisual integration, as measured by the McGurk effect. All experiments employed a repeated-measures factorial design. For the audiovisual asynchrony manipulation, the soundtrack could be shifted forwards or backwards in time relative to the visual sequence over a range of ±500 msec through nine equal steps of 125 msec including zero (sound synchronous with video). In Experiments 1 and 2, an independent variable was the congruency of lip-movements with voice (see Stimuli above). There were two possible lip-voice combinations for each congruent/incongruent pairing. Only incongruous conditions were used for assessing McGurk interference. Two dependent measures were obtained from two responses elicited after each trial, for TOJs and phoneme identity/stream–bounce judgements respectively.

### 3.4 Procedure

Each trial began with a fixation display. Following a keypress and a blank interval (duration randomly selected from the range 1000 ± 500 msec), a movie was displayed for 2800 msec. On each trial the audiovisual asynchrony and stimulus pairing were selected pseudo-randomly. Each stimulus pairing was presented at each of the nine possible asynchronies 8–10 times in pseudorandom order. Following movie offset, there were two successive forced-choice questions. Firstly, a TOJ task asked whether the voice (or beep) onset preceded or followed the lip-movement (or visual collision). In Experiments 1 and 2, the second question elicited a phoneme discrimination, asking whether the voice said “ba” or “da” [a third option for ‘other’, used on only .3% ± .3% standard error of the mean (SEM) of trials, was not included in further analysis]. Subjects were encouraged to choose the option that sounded the closest to what they heard. In Experiment 3, this second question asked subjects to indicate whether they saw the balls bounce or stream through each other. The additional tests performed by PH, with finger-clicks, flashes and noise-bursts, and scrambled speech, were all run as a single-task eliciting TOJs.

### 3.5 Analysis

For TOJ, we plotted the proportion of ‘voice second’ responses (where the auditory onset was judged to lag the visual onset) as a psychometric function of actual auditory lag time in milliseconds (note that negative lag denotes an auditory lead). The proportion of ‘sound second’ values was typically below 50% for negative auditory lags (i.e., sound leads vision), and above 50% for positive auditory lags. A logistic function was then fitted to the psychometric data, using a maximum-likelihood algorithm provided by the PSIGNIFIT toolbox for Matlab (Wichmann and Hill, 2001). We could then read off from the fitted function the critical auditory lag corresponding to the participant's PSS. This is the point at which the participant is at chance (50%) deciding whether the sound came first or second relative to the visual onset. The same software was used to find the slope of the function and to derive 95% confidence intervals for both PSS and slope estimates, via a bootstrapping procedure. Finally, we estimated the additional auditory lag required for the participant to go from responding at chance to responding ‘voice second’ 75% of the time. The resulting value quantifies the lag that can produce a Just Noticeable Difference (JND) between subjectively synchronous and asynchronous stimuli.

For the phoneme discrimination task we obtained the proportion of trials in which the reported phoneme was consistent with the lip-movements, averaged across incongruous conditions only. For example, a ‘ba’ response to /da/ + [ba] and a ‘da’ response to /ba/ + [ga] were scored as ‘consistent’. This was plotted as a psychometric function of auditory lag. The data from each of the two incongruent conditions, plus the average across them, were fit using an asymmetric double sigmoid function (ADS, following van Wassenhove et al., 2007), which results in a bell-shaped curve with adjustable height, width and asymmetry, using the following equation:

$$y=\frac{1}{2}\left[\tanh\left(\frac{x-c_{1}}{w_{1}}\right)-\tanh\left(\frac{x-c_{2}}{w_{2}}\right)\right]\;\text{with}\;\text{constraints}\;w_{1}>0\;\text{and}\;w_{2}>0$$

The optimal auditory lag for maximum McGurk interference (tMcG) from vision was read off at the peak of each of these interpolated functions and averaged, with 95% confidence intervals derived from fits of 1000 bootstrapped samples. For stream–bounce judgements, ADS functions were fitted to the proportion of ‘bounce’ responses.

Across subjects, mean (and SD) of *R*^2^ values for goodness of fit of functions to the psychometric data were .89 (.13) for the TOJ task, and .75 (.18) for the phoneme discrimination task.

## 4 Results

All inferential statistics reported in the following are based on parametric statistics, as data did not deviate significantly from normality (Kolmogorov–Smirnov *p* > .05).

### 4.1 Experiment 1

#### 4.1.1 PH

**Fig. 3:**
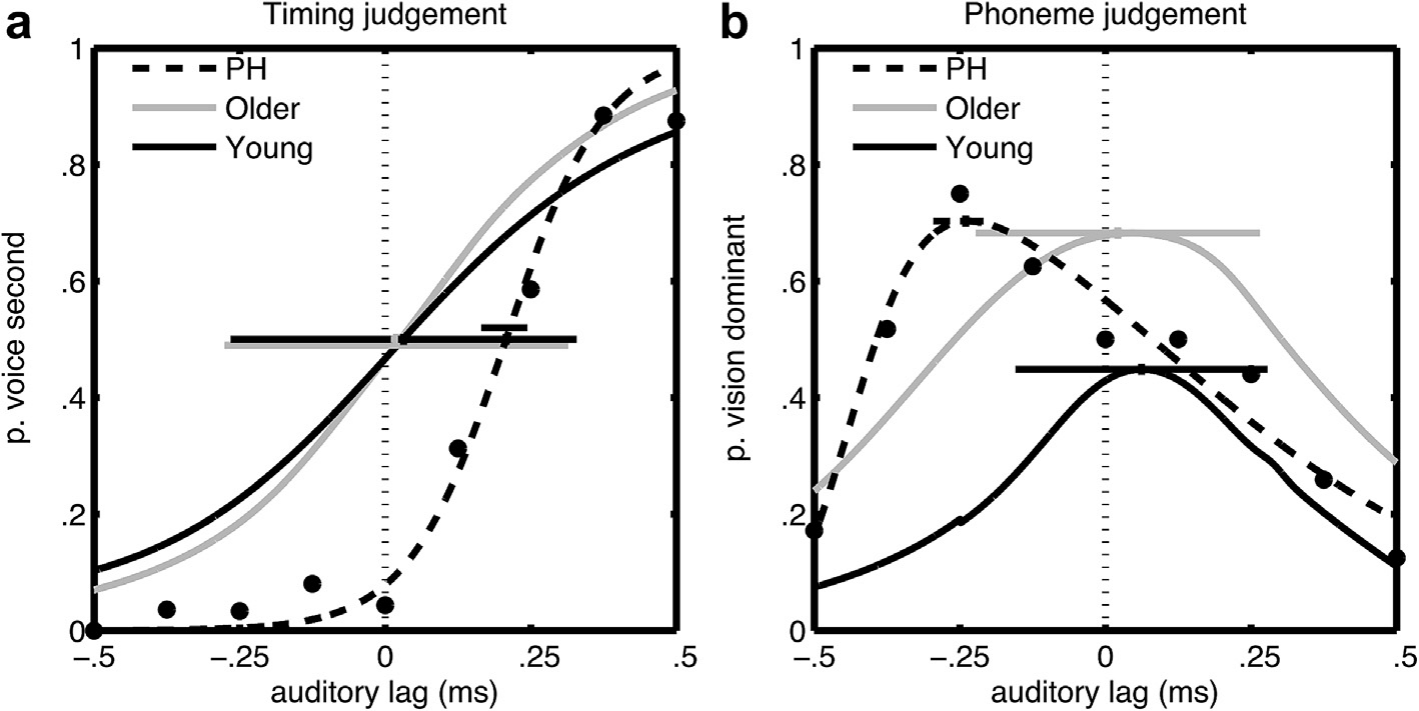
Psychometric data for PH (with black data points and interpolation using a broken line), and for healthy young (black continuous function) and older (grey) groups. a) TOJ: proportion of ‘voice second’ reports (*y*-axis) for different auditory lags (negative *x*-values for auditory lead), interpolated with a logistic function. Horizontals indicate the PSS with 95% confidence intervals based on bootstrapped estimates for PH and on SEMean for controls. b) Phoneme discrimination task: proportion of responses following lip-movement, averaged across incongruous conditions only, interpolated using ADS functions. Auditory lag for tMcG was read off at the maximum.

PH's TOJs corroborated his subjective report of voice leading lips. His PSS was shifted away from veridical to 210 msec auditory lag. This means that subjective synchrony could only be restored for PH by artificially lagging voices relative to lip-movements (by 210 msec, see Table 2), at which point temporal order became indistinguishable (Fig. 3a). Also very curiously, the optimal asynchrony for maximum McGurk (tMcG) showed almost exactly the opposite asynchrony (240 msec auditory lead was required for optimum McGurk). Thus voices effectively lagged lip-movements for the purposes of audiovisual speech integration (Fig. 3b).

To investigate the generality of PH's auditory lead we tested him on a variety of biological and artificial non-speech stimuli, using single-task TOJs. In separate single-task tests, we found PSS was closer to veridical for artificial stimuli (flash/noise-burst pairs: 2 ± 99 msec auditory lag, 95% confidence interval), finger-clicks (with no significant difference between movies beginning from open or closed-hand positions: 64 ± 85 msec), and for unintelligible noise-vocoded speech (52 ± 98 msec). In contrast, a similar single-task paradigm with the original speech stimuli showed a similar PSS shift as in the dual-task situation (210 ± 90 msec). It may therefore be concluded that PH's PSS shift was specific to speech, and not dependent on the number of concurrent tasks.

#### 4.1.2 Comparison with controls

How unusual is PH? Using a modified *t* test for comparing an individual's test score with a small normative sample (Crawford and Howell, 1998), we found PH's tMcG was significantly greater than for 10 healthy age-matched participants [Crawford *t*(9) = 2.23, *p* = .05]. The discrepancy between PH's PSS and tMcG measures was also significantly greater than for the control sample [Crawford *t*(9) = 2.46, *p* = .04]. On these measures PH therefore does seem abnormal. However his PSS was not significantly deviant from controls [*t*(9) = 1.50, *p* = .17 ] (Table 2). Fig. 3 illustrates these results graphically as psychometric functions for PH compared with the group average function.

We repeated the analysis after collecting data from a further sample of 27 young participants (see Expt. 2) with similar results (Table 2). Relative to the tMcG measure, PH was again significantly deviant from young participants [*t*(25) = 2.64, *p* = .01], and from the whole combined-age sample [*t*(35) = 2.55, *p* = .02]. The discrepancy between PSS and tMcG measures was also significant for the young [*t*(25) = 2.14, *p* = .04] and combined-age sample [*t*(35) = 2.25, *p* = .03]. However, he was not deviant relative to the PSS for young [*t*(25) = 1.28, *p* = .21] and the combined-age sample [*t*(35) = 1.37, *p* = .18].

**Fig. 4:**
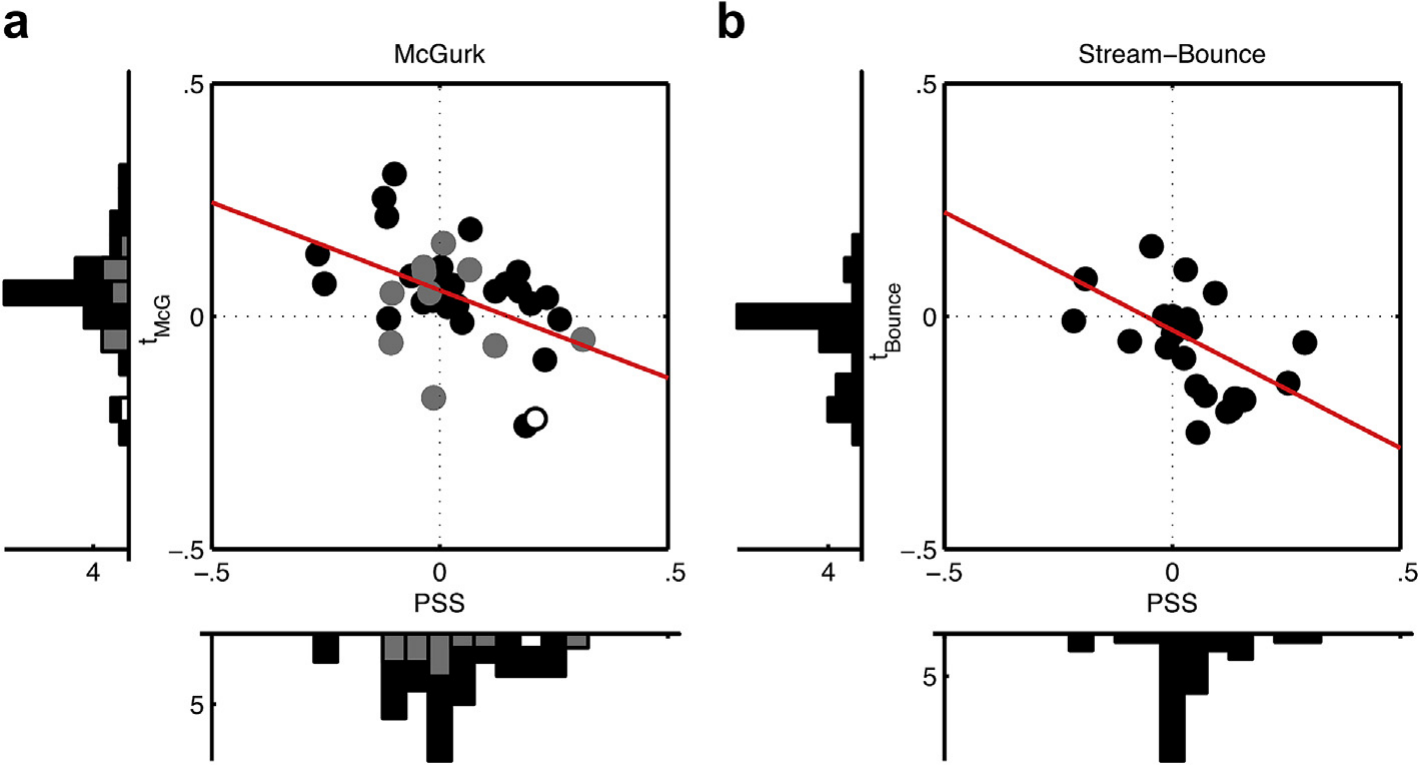
PSS (*x*-axis) plotted against asynchrony for maximum a) McGurk, i.e., tMcG (open circle: PH; grey: older; black: young) and b) bounce illusions (tBounce) with line of best fit, and marginal histograms.

It is surprising to note that on the measure that reflects PH's subjective report of voice leading lips, some healthy participants showed PSS values of comparable magnitude to PH (Fig. 4a). Given that some normal participants seemed to show a similar magnitude of PSS shift, is PH is the only one aware of asynchrony? 10/37 participants consistently reported a visual or auditory lead on more than 75% of synchronous trials. Thus for these participants, the difference between veridically synchronous stimuli and their personal PSS was actually greater than their JND for perceiving asynchrony. In other words, these subjects seemed to reliably perceive physically synchronous stimuli as asynchronous, at least under laboratory conditions.

### 4.2 Experiment 2: McGurk with normative sample

PH's two lesions in pons and STN seem well placed to disrupt audition and/or timing (Halverson and Freeman, 2010; Kolomiets et al., 2001; Teki et al., 2011), and might explain the auditory lagging observed in tMcG. But how could the same lesions also produce an opposite shift in PSS, and PH's corresponding experience of auditory leading?

It may be instructive to note that in PH our two measures of sensory timing are distributed roughly symmetrically around zero auditory lag. Thus despite the temporal disparity between mechanisms it seems that on average across measures he can still achieve near-veridical timing (see General discussion for further elaboration). It is suggested that in order to maintain veridical performance, and thus continue to live in the ‘present moment’, pathological auditory slowing within impaired mechanisms is balanced by perceiving auditory timing in preserved mechanisms as slightly earlier than veridical. In other words the asynchronies obtained within each mechanism might have been renormalised relative to the average asynchrony across mechanisms.

Such renormalisation might explain how veridical perception is maintained on average following pathological disruption of timing in selected mechanisms, but for neurologically healthy people the prediction is highly counterintuitive: individual differences (Stone et al., 2001) which bias one measure of subjective timing in one direction (e.g., auditory lead for PSS) might be associated with the opposite bias in other measures (e.g., auditory lag for tMcG, or vice versa). This prediction of a negative correlation contrasts with the positive correlation predicted if synchronising mechanisms brought individual differences in PSS (Stone et al., 2001) and tMcG into agreement (Fujisaki et al., 2004; Harris et al., 2008; Spence and Squire, 2003; Vroomen and Keetels, 2010).

To test this we measured the correlation between PSS and tMcG, across the whole sample of young and older participants (total *N* = 37). As predicted by the compensation hypothesis above, the correlation was significantly negative (*N* = 38, Pearson's *ρ* = −.47, *p* = .003, Fig. 4a). Yet on average performance on both measures remained near-veridical (Fig. 3).

### 4.3 Experiment 3: Stream–Bounce

Is this apparent repulsion of timing measures just a speech-specific phenomenon? We tested this with the Stream–Bounce illusion (Sekuler et al., 1997, Fig. 1), in which two approaching ‘balls’ may appear to bounce off each other when their collision coincides with a sound, rather than streaming past each other. As before, there were two questions after each trial. The first probed the temporal order of the sound relative to the visual collision. The second required participants to judge whether they saw the balls bouncing off each other or streaming through each other, from which we estimated the asynchrony for maximum ‘bounce’ (tBounce). We again found a negative correlation between PSS and tBounce (Pearson's *ρ* = −.54, *p* = .001, for 24 new young participants, Fig. 4b). Note that in contrast to the McGurk illusion for speech where vision influences hearing, in this non-speech illusion, hearing influences vision. Thus we may infer that this negative correlation pattern, replicated for speech and non-speech, and in both directions of audiovisual influence, reflects a general (rather than a stimulus-specific or task-specific) characteristic of perception.

## 5 General discussion

Our psychophysical investigation of PH confirmed his subjective report of lips lagging voices, when measured using TOJ, but revealed the opposite bias for McGurk integration, with temporal tuning favouring auditory lagging. Thus PH suffers not only from an acquired disruption of synchronisation, but also a violation of perceptual unity of timing across different aspects of the same pairing of auditory and visual stimuli. Neurologically normal individuals also showed a comparable opponency between our two measures (in speech and non-speech and in both directions of audiovisual influence): thus if one subject showed auditory lagging for TOJ, the McGurk measure tended to show auditory leading (or vice versa). Altogether, these counterintuitive findings suggest that perception of synchrony and integration depend on distinct rather than common synchronising mechanisms, and reveal one strategy by which the brain might achieve near-veridical perception of the timing of multisensory events, at least on average, despite the evident temporal disunity of sensory processing.

### 5.1 How unusual is PH?

If specialised mechanisms existed to synchronise senses in normal brains, one would expect to find more cases of acquired sensory desynchronisation when such mechanisms are lesioned (Wiener et al., 2011). There has only been one previous report, of patient AWF (Hamilton et al., 2006). However the similarity with PH is difficult to assess, as the direction of AWF's acquired ‘temporal mismatch’ was not specified, and he was only tested with synchronous stimuli. AWF showed no McGurk effect while PH did when tested with asynchronous (auditory leading) stimuli. AWF's lesions are also in a quite different location, in right parietal cortex, while PH's lesions are in mid-brain and brainstem. We can at least claim that the present case is the first to be reported of an acquired subjective auditory lead, which is speech-specific and accompanied by an auditory lag for optimal McGurk integration.

Surprisingly, some healthy participants also showed large deviations of PSS; indeed for some, synchronous stimuli were just-noticeably asynchronous. Thus it seems PH is not so unusual in terms of experiencing a mismatch in audiovisual timing. Such ubiquitous sensory asynchrony further undermines support for the existence of specialised synchronisation mechanisms. It also raises the obvious question of why only PH is aware of his asynchrony in his everyday life. It is possible that our TOJ results from normal participants are specific to our laboratory conditions. In the outside world we learn to expect that when auditory and visual events originate from the same source, they are also very likely to occur simultaneously, regardless of their sensory timing. Under this unity assumption (Vatakis and Spence, 2007; Welch and Warren, 1980) our perception might tend to rely more on this expectation than any sensory evidence of asynchrony. Our paradigm, by contrast, presented a randomised range of asynchronous stimuli with no feedback about which was actually synchronous. In this situation the unity assumption cannot be confidently applied, and perceptions may rely more on asynchronous sensory inputs than prior expectations. Under such conditions even neurologically healthy subjects might notice an asynchrony given actually synchronous stimuli. As for PH, his subjective asynchrony (which changed unexpectedly later in life) might just be too great for him to reconcile with the assumption of unity, even outside the lab (Vatakis and Spence, 2007; Welch and Warren, 1980).

While PH's auditory lead for PSS is not statistically abnormal, his auditory lag for optimal McGurk (tMcG) is. This might be explained if the principle impairment caused by his lesions is actually a slowing of auditory processing, consistent with the location of his lesion on a tract connecting with the inferior colliculus, part of the early auditory system (see Supplementary Materials for an analysis of tractography).

The dissociation between PH's temporal tuning of subjective simultaneity for TOJ, versus for phoneme discrimination, suggests that each different task may probe different mechanisms, each subject to their own neural asynchronies (Aschersleben and Prinz, 1995). For example, one mechanism might be involved in speech integration and the other in judging sensory synchrony (Calvert, 2001; Miller and D'Esposito, 2005; Vroomen and Stekelenburg, 2011). The further dissociation between PSS for speech versus non-speech would be consistent with the existence of special mechanisms for these different stimulus types (Vatakis et al., 2008). Alternatively the same mechanisms might have different temporal tunings depending on the low-level characteristics of the specific stimulus presented (Vroomen and Stekelenburg, 2011). From these dissociations it seems, at least for PH, that there are indeed multiple clocks (see Introduction), whose discrepant timings cannot be reconciled.

### 5.2 How closely do measures of integration normally correspond with measures of synchrony?

An appealing intuition is that single physical events should be associated with a unitary percept (Welch and Warren, 1980). Evidence suggests that the brain strives for (Vatakis and Spence, 2007), and benefits from (Soto-Faraco and Alsius, 2007, 2009; van Wassenhove et al., 2007) such unity. But PH shows a dramatic failure of unity, with voices subjectively leading lip-movements, at the same time as effectively lagging lip-movements for the purposes of integration. Is PH just an exception to the putative rule that unity is normally achieved? Previous studies with normal participants (using the original paradigm borrowed here) have also reported ‘dual perception’ of good lip-voice integration despite a detectable audiovisual asynchrony (Soto-Faraco and Alsius, 2007). However such violations were small when measured on average across participants, and could arguably have reflected different decision criteria for the two concurrent judgements. The TOJ task may be particularly susceptible to response biases (García-Pérez and Alcalá-Quintana, 2012; Soto-Faraco and Alsius, 2009; van Eijk et al., 2008). However such criterion or response bias effects, or attentional biases such as prior-entry (Spence and Parise, 2010; Spence et al., 2001) cannot easily explain away the negative correlation we show in Fig. 4 (see our Supplementary Discussion). Our analysis of individual differences reveals the true extent to which subjective unity is routinely violated in normal participants, who can sometimes perceive, concurrently, different aspects of a single pair of auditory and visual events to be occurring at quite different times relative to each other.

### 5.3 Theoretical accounts

Over the years there have been a variety of approaches to the problem of how temporal unity can be maintained across asynchronous processes in the brain (Keetels and Vroomen, 2012). One solution might be to have dedicated mechanisms for timing events, via a supramodal mechanism (Hanson et al., 2008; Treisman, 1963), or specialised timing mechanisms residing in cerebellum or basal ganglia (Ivry and Spencer, 2004), functioning to provide a common time code for multisensory events. Timing discrepancies might also be minimised (Keetels and Vroomen, 2012), via temporal ventriloquism (Freeman and Driver, 2008; Morein-Zamir et al., 2003; Vroomen and De Gelder, 2004), or by selectively delaying one modality (Sternberg and Knoll, 1973), or by recalibration of temporal codes (Fujisaki et al., 2004), so that a frequently occurring neural asynchrony is perceived as synchronous. Compensatory adjustments might also be made in a context-sensitive way, for example taking into account the distance of events from the observer (Harris et al., 2008) or the prior likelihood that the causal events are actually synchronous or not (Miyazaki et al., 2006; Yamamoto et al., 2012).

**Fig. 5:**
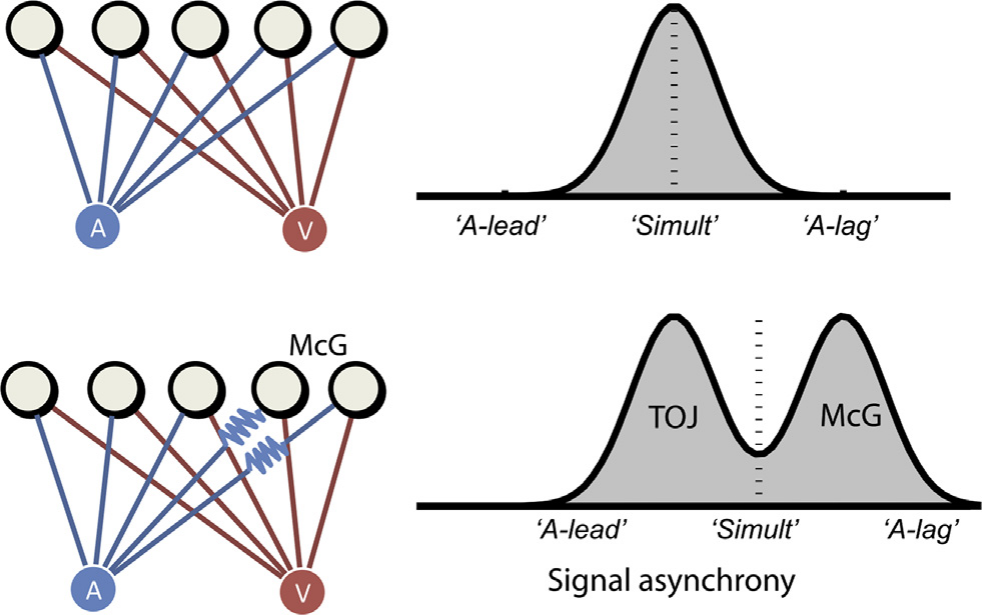
Temporal renormalisation theory: hypothetical relationship between neural and subjective audiovisual asynchrony. Top left: signals from synchronous auditory and visual stimuli (represented by blue and red disks) converge on different audiovisual mechanisms in the brain via different routes (grey disks). For individual mechanisms the actual stimulus timing cannot be dissociated from the propagation latency. Top right: schematic of the evoked distribution of neural asynchronies, across mechanisms, plotting probability of different asynchronies, as a function of neural asynchrony, with increasing delays of auditory signals relative to visual towards the right. The *x*-axis text refers to the *subjective* experience of auditory lead, simultaneity, or auditory lag, given these different neural asynchronies. The neural asynchrony at the central tendency of the distribution is the one which relates most reliably to the objective timing of the auditory and visual stimuli, after delays within individual mechanisms have been averaged out. Following experience with this distribution in natural contexts where objective synchrony is likely, tasks probing mechanisms registering asynchronies near this average may evoke perception of synchrony (marked with a dotted line and ‘Simult’); asynchronies registered within other mechanisms are perceived in proportion to their distance from the average. Lower left: an example where auditory inputs to a subset of mechanisms (towards the right) are particularly delayed. For patient PH it is assumed that these mechanisms contribute to the temporal tuning of the McGurk illusion (labelled McG; see main text), while mechanisms involved in TOJ are preserved. Lower right: the bimodal distribution resulting from delayed auditory input for the McGurk task. The mean of the distribution has shifted towards the auditory-lagged mechanisms serving the McGurk task (labelled McG). The perceived asynchrony within each mechanism is renormalised to this new distribution mean. The result is that neural asynchronies for unaffected mechanisms (here labelled TOJ) originally perceived as synchronous (as in the top example) are now perceived as auditory leading.

The above accounts, on first sight, seem difficult to square with the present evidence of disunity, and particularly the negative correlation between different measures of audiovisual timing (Fig. 4). Our results suggest that timing discrepancies between mechanisms serving performance of our synchronisation and integration tasks cannot be fully reconciled. However, as we explain below (and in Fig. 5), our evidence is still consistent with the mainstream assumption that the brain adjusts for differences in neural timing between distinct modalities. Our account just makes explicit the assumption that this adjustment is made based on *average* differences in timing: either between modalities (Harris et al., 2008), or in principle more generally between cognitive processes or any arbitrary groupings of temporally discrepant mechanisms.

### 5.4 Temporal renormalisation

Given the present evidence that disparities in timing for different tasks cannot be fully minimised, there appears to be no escape from the multiple-clocks problem: ‘*with one clock you always know the time; with two you are never sure*’. But of course, Segal's maxim is misleading. Given a room full of clocks, each independently subject to inaccuracies, our best guess at the correct time comes from the average across all clocks. Thus statistically, the more clocks we have the better for accurately estimating this average. Such averaging may be how the brain solves the multiple-clocks problem. This problem is that different auditory and visual stimuli are processed at different speeds, and arrive at different mechanisms (e.g., contributing to synchrony and integration judgements respectively) at different times, resulting in a distribution of neural timings measured across the different mechanisms. From the point of view of an individual mechanism contributing to this distribution, it is uncertain to what extent the timing of its inputs reflects the true external timing of events or just internal processing delays (Scharnowski et al., 2013). But the average over the distribution provides a purer estimate of the neural timing that relates most reliably to the true timing of external events (see Fig. 5 for a schematic illustration, and Supplementary Discussion of how this could apply before and/or after unimodal signals). We propose that *discrepancies in timing between mechanisms are not minimised but perceived relative to their average timing*.

In contrast to the other theoretical alternatives, this temporal renormalisation theory provides a fuller and more explicit account of all of our paradoxical findings: why a lesion produces opposite lags in different measures; why in normal participants different measures of subjective timing appear mutually repulsive, and how despite such disunity perception remains near-veridical *on average across measures*. To see how these phenomena emerge, note that in the multiple-clocks analogy, if one clock is particularly slow then this will bias the average, relative to which even the correct clocks will seem to be fast. In the brain, the mean neural delay of each sensory modality could also be attracted to particularly slow (or fast) neural events such that even events with relatively normal timing may be perceived as slightly fast (or slow). In PH, the integrative mechanisms probed by the McGurk task may have an unusually delayed auditory input, due to a selective brain lesion. The central tendency of the distribution will shift towards auditory lags, and relative to this, auditory signals from other unaffected mechanisms, such as those performing TOJ, will now be perceived to be leading. Yet on average across these measures, and despite pathological disruptions of timing, performance remains near-veridical. Renormalisation also explains the negative correlation we observed in healthy individuals, for whom auditory and visual timing may vary naturally in a similar (or opposite) direction to PH: in different people the greater the deviation in the auditory lead (lag) direction for some mechanisms, the more auditory leading (lagging) will be reported for other mechanisms, relative to the mean asynchrony, thus resulting in an *apparent* antagonism between mechanisms. Given that the mean neural asynchrony most reliably relates to external synchrony (under the unity assumption), renormalisation explains how near-veridical performance is maintained on average, across mechanisms and also across subjects.

### 5.5 Simulation

Computations based on statistical distributions are routinely proposed in Bayesian theories of perception (Miyazaki et al., 2006; Yamamoto et al., 2012), while functions similar to averaging over such distributions have been considered in theories of population coding (Roach et al., 2011). Assuming similar mechanisms in principle, we performed a simple simulation, in which we plotted values sampled from two random variables (‘clocks’), after subtracting each from the average across a population of clocks. We found that this simple renormalisation model could accurately simulate the negative correlation observed (see Supplementary Methods S2 and Supplementary Figure 2 for further details). This serves to demonstrate how the observed negative correlation phenomenon might emerge simply as a consequence of renormalisation, and *not* due to any explicit antagonism between mechanisms.

### 5.6 Conclusions

Neuroscientists and philosophers have long pondered the relationship between subjective and neural timing (Dennett and Kinsbourne, 1995; Harris et al., 2008; Spence and Squire, 2003; Zeki and Bartels, 1998). Our observations with PH and with neurologically healthy participants confirm that perception is characterised fundamentally by asynchrony and disunity: different aspects of the same pair of multisensory stimuli may be perceived with different asynchronies, and these discrepancies cannot be fully minimised. But an apparent antagonism between complementary measures of subjective timing reveals a superordinate principle, by which discrepant timings in the brain may nevertheless be renormalised to their average neural timing. By relating subjective timing to average neural timing, temporal renormalisation explains (1) why after a lesion PH experiences auditory leading in one task but the opposite auditory lead in another, (2) why different timing measures are negatively correlated across normal individuals, and (3) how the brain might tell the time from multiple clocks, with near-veridical accuracy, without needing resynchronising mechanisms.

## Figures and Tables

**Table 1:** Neuropsychological test results for PH.

Test	PH
Wechsler abbreviated scale of intelligence (The Psychological Corporation, 1999)	
Full scale IQ	136
Verbal IQ	133
Performance IQ	129

Test of everyday attention	
Elevator counting	6/7
Elevator counting with distraction	10/10

Visual Object and Space Perception Battery (Warrington and James, 1991)	
Shape detection	20/20
Incomplete Letters	20/20
Silhouettes	19/30
Object decision	19/20
Dot counting	10/10
Position discrimination	20/20
Number location	9/10
Cube analysis	10/10

Sentence repetition (auditory only)	22/22
Low + high frequency word repetition	100%

Praxis	Normal

**Table 2:** Mean results from McGurk experiment.

	*N*	PSS^a^	CI^b^	JND^c^	CI	tMcG^d^	CI
PH	1	210	±40	93	±35	−240	±56
Older	10	19	±94	176	±64	21	±78
Younger	27	31	±57	272	±81	62	±42

a PSS, in milliseconds; positive values for auditory lag, negative for auditory lead.

b CI: 95% confidence interval (msec); estimated for PH by bootstrapping and for controls from SEMean.

c JND from subjective simultaneity (msec).

d tMcG: asynchrony for maximum McGurk effect (msec). Positive values for auditory lag.
