# Supplementary figures and images for: Sight and sound out of synch: Fragmentation and renormalisation of audiovisual integration and subjective timing
Source: Cortex. 2013 Nov;49(10):2875–87. doi: 10.1016/j.cortex.2013.03.006 (PMC3878386; doi:10.1016/j.cortex.2013.03.006)

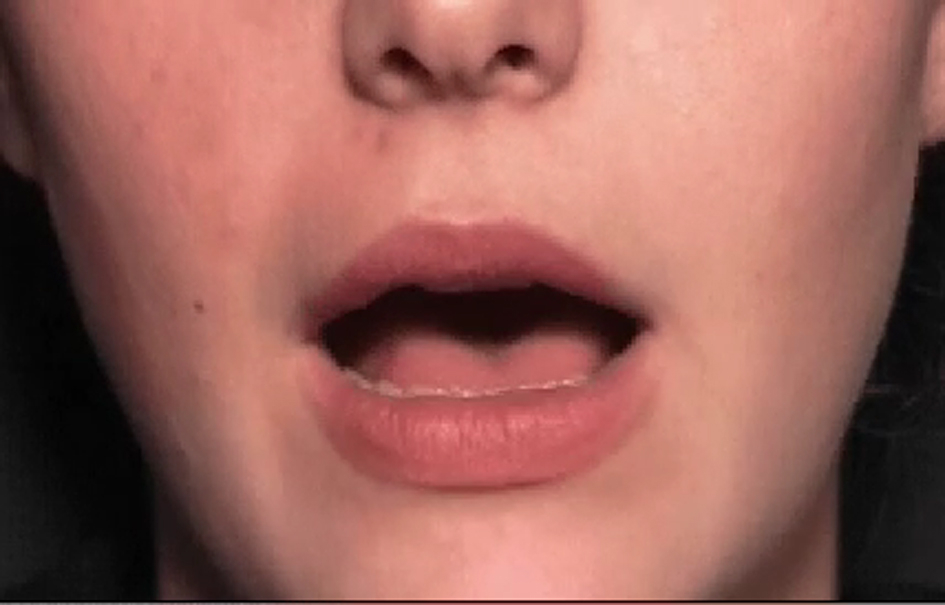

Supplement: Video 1 — McGurk stimulus demo: Four combinations, played consecutively: 1. Auditory /ba/ with visual [ba]: congruent. 2. Auditory /ba/ with visual [ga] (incongruent: McGurk effect sounds like “da”). 3. Auditory /da/ with visual [ba] (incongruent: McGurk effect sounds like “ba”). 4. Auditory /da/ with visual [da]: congruent. [file mmc2.jpg]

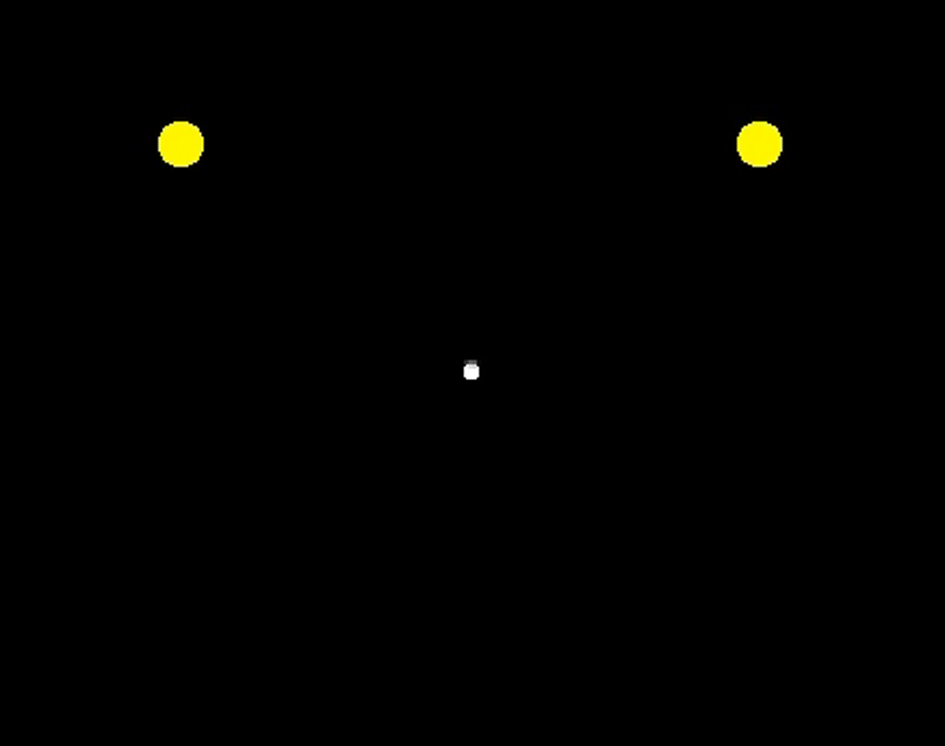

Supplement: Video 2 — Stream–bounce stimulus demo. Two examples, played consecutively: 1. Beep simultaneous with visual collision. 2. Beep lags visual collision by 150 msec. [file mmc3.jpg]
