# Supplementary material for: Sight and sound out of synch: Fragmentation and renormalisation of audiovisual integration and subjective timing
Source: Cortex. 2013 Nov;49(10):2875–87. doi: 10.1016/j.cortex.2013.03.006 (PMC3878386; doi:10.1016/j.cortex.2013.03.006)
Supplement: Supplementary file 3 [file mmc1.docx]

**Supplementary Materials
Freeman et al: Sight and sound out of synch.**

Table of Contents

S1. Diffusion Tensor Imaging 1

Supplementary Figure 1: T2 weighted images with DTI tracts marked in green and lesion 2 in red in panel 3 (left pons). 2

S2. Supplementary Discussion of decisional or attentional interpretations 3

S3. Supplementary Discussion of renormalisation theory 4

S4. Simulation of renormalisation model 5

Supplementary Figure 2: Simulation results 6

# Diffusion Tensor Imaging

Diffusion tensor imaging (DTI) was undertaken using images from healthy subjects, to identify brain regions which are connected to the lesion sites. Brain images from six neurologically normal right-handed males were registered together using DARTEL (in part of SPM toolbox for Matlab, [www.fil.ion.ucl.ac.uk/spm](http://www.fil.ion.ucl.ac.uk/spm)). Lesion masks were delineated by first defining a sphere around each of the patient’s lesions, and then applying an intensity threshold to define the actual lesion ROI in a non-biased fashion. These were warped to 1mm isotropic MNI space and then back projected to individual healthy subject space. Lesion 1 was moved to the individual healthy subject space using DARTEL inverse warp. Lesion 2 was moved to standard space using the SUIT toolbox (optimised for cerebello-brainstem registration). Tractography was performed from every individual voxel in subject space using FSL ProbtrackX (<http://www.fmrib.ox.ac.uk/fsl/fdt/fdt_probtrackx.html>). Each voxel was sampled 5000 times, with curvature threshold corresponding to a minimum angle of 80 degrees. Individual raw diffusion profiles were thresholded at 1% and binarised. All were moved back to standard space, and averaged.

## Supplementary Figure 1: T2 weighted images with DTI tracts marked in green and lesion 2 in red in panel 3 (left pons).

# Supplementary Discussion of decisional or attentional interpretations

In Soto-Faraco & Alsius (2007), discrepancies between measures might arguably have reflected differences in criterion for the two concurrent judgements. Could apparent disunity between our two measures of subjective timing also be explained in terms of differences in decision criteria assigned to different tasks? This class of explanation has been used to account for discrepancies between different measures of PSS, obtained from temporal order judgements and simultaneity judgements (García-Pérez and Alcalá-Quintana, 2012; Schneider and Bavelier, 2003; Van Eijk, Kohlrausch, Juola, and Van De Par, 2008), which show a null correlation (Van Eijk et al., 2008). Individual variability could also be caused by differences in weighting of attention to vision versus audition, causing prior entry effects (Spence and Parise, 2010; Spence, Shore, and Klein, 2001). But while it is easy to imagine how inconsistent decisional or attentional biases could obscure a positive correlation between two complementary measures, it is harder to explain how biases could result in a negative correlation. Considering the dual-task context of the just Stream-Bounce experiment for now, two possible forms of bias may be distinguished: firstly, a bias which results in an unequal tendency to choose one of two possible responses, and secondly, a bias in which the second response is contingent on the first. Considering the first kind, responses might be biased, for example, towards the ‘voice second’ and ‘bounce’ responses. This would shift the psychometric function for TOJ horizontally, resulting PSS values biased towards greater auditory lag. However the same response bias could only increase the height of the ‘bounce‘ function, and could not shift the location of its peak along the asynchrony axis. With the second response-contingent type of bias, subjects might tend to press the same key twice for both tasks, or even tend to switch to the opposite key for the second keypress. This could increase or decrease the proportion of ‘bounce’ responses as a function of auditory lag, resulting in a shift of tBounce values, but would have no effect on PSS (as this is the first response upon which the second is contingent). It can therefore be seen that neither of these two biases alone would result in a negative correlation. Only the specific combination of the first bias, which shifts PSS, with the second bias towards making opposite responses for the second question, thus shifting tBounce in the opposite direction, could in principle create a negative correlation. The situation is even more complicated in the McGurk experiment, in which the direction of the second bias would have to depend on the specific stimulus context, because the McGurk illusion is classified by ‘ba‘ responses when the actual stimulus is /da/ is heard and ‘da‘ responses when the actual auditory stimulus is /ba/. However by the nature of this illusion, such perceptual discrimination is by definition extremely difficult. In conclusion, a complete explanation based on response biases may arguably be ruled out on grounds of implausibility and parsimony.

# Supplementary Discussion of renormalisation theory

Temporal renormalisation could be applied before and/or after unimodal signals are combined, given different assumptions. In the unimodal case, the average time of auditory neural events could be computed separately from visual, and a compensatory adjustment made to the perceived timing so that signals whose timings are close to their average are perceived as happening *'now'*. This unimodal adjustment would have to be propagated to the stage of multimodal convergence, so that unimodal signals whose timings are close to their unimodal average are perceived as synchronous. Those signals further from the average in either modality would then be perceived as asynchronous. However this kind of unimodal compensation would not help to compensate for audiovisual asynchronies resulting from different propagation latencies as signals converge on different multimodal mechanisms.

An alternative more general formulation might assume that adjustments are made after signals are combined, forming a distribution of audiovisual asynchronies. In natural contexts where most multisensory events occurring within a short interval can be generally assumed to originate from a synchronous source (i.e. the unity assumption), the evoked distribution of internal asynchronies could be used to construct a window of simultaneity: asynchronies close to the centroid of the distribution will be perceived as simultaneous; conversely outlying asynchronies (relative to the width of the distribution) may be more likely to be caused by truly asynchronous external events, and perceived as such. An advantage of this scheme is that the window of simultaneity could be constructed fairly rapidly, on the basis of the peak and spread of arbitrary distributions resulting from any combination of stimuli at any observer distance. This therefore provides a mechanism for achieving simultaneity constancy (at least at a first approximation) while avoiding the potential complexities of having to learn the specific internal asynchronies evoked by potentially many different stimuli in many different contexts (Harris, Harrar, Jaekl, and Kopinska, 2008).

# Simulation of renormalisation model

To see whether temporal renormalisation could explain the negative correlation observed, we performed a simple simulation. We generated two noisy 'clocks' by randomly sampling from a normal distribution with the same N, means and standard deviations as each of the original PSS and tMcG datasets. The renormalisation idea assumes that asynchronies measured by each clock are each subtracted from the average across the distribution of all clocks. Clearly, if there were only two independent clocks, the result would be a perfect negative correlation, with a regression slope of -1. We therefore tested how the slope depended on adding further independently noisy variables to the distribution, with the statistics derived from the combination of our two original datasets. These could represent additional independent clocks, which were not probed by either of our two tasks, or just general noisiness of perception or responses. It was sufficient to add just one additional variable to simulate the results we obtained and to obtain a similar regression slope. Supplementary Figure 3a shows an example of one such simulated datasets, and Supplementary Figure 3b shows the regression slope as a function of number of additional noisy variables, with shaded regions indicating 95% confidence intervals based on 1000 simulations. The horizontal line indicates the slope obtained with 38 participants in the original McGurk experiment.

## Supplementary Figure 2: Simulation results

See text above for details.

# References

García-Pérez MA, and Alcalá-Quintana R. On the discrepant results in synchrony judgment and temporal-order judgment tasks: a quantitative model. *Psychonomic Bulletin & Review*, *19* (5):820–846, 2012.

Harris LR, Harrar V, Jaekl P, and Kopinska A. Mechanisms of simultaneity constancy. In Nijhawan R (Ed), *Issues of Space and Time in Perception and Action* (pp. 232–253). Cambridge University Press, 2008.

Schneider KA, and Bavelier D. Components of visual prior entry. *Cognitive Psychology*, *47* (4):333–366, 2003.

Spence C, and Parise C. Prior-entry: a review. *Consciousness and cognition*, *19* (1):364–379, 2010.

Spence C, Shore DI, and Klein RM. Multisensory prior entry. *Journal of Experimental Psychology: General*, *130* (4):799–832, 2001.

Van Eijk RLJ, Kohlrausch A, Juola JF, and Van De Par S. Audiovisual synchrony and temporal order judgments: effects of experimental method and stimulus type. *Perception & Psychophysics*, *70* (6):955–968, 2008.
